# Global research trends in AI-assisted blood glucose management: a bibliometric study

**DOI:** 10.3389/fendo.2025.1579640

**Published:** 2025-05-28

**Authors:** Li Yuan, Yangtian Wang, Meiping Xing, Tao Liu, Dan Xiang

**Affiliations:** ^1^ Department of Endocrinology, Nanjing University Medical School Affiliated Taikang Xianlin Drum Tower Hospital, Nanjing, China; ^2^ School of Chinese Medicine, Nanjing University of Chinese Medicine, Nanjing, China

**Keywords:** AI, blood glucose management, diabetes, machine learning, continuous glucose monitoring

## Abstract

**Background:**

AI-assisted blood glucose management has become a promising method to enhance diabetes care, leveraging technologies like continuous glucose monitoring (CGM) and predictive models. A comprehensive bibliometric analysis is needed to understand the evolving trends in this research area.

**Methods:**

A bibliometric analysis was performed on 482 articles from the Web of Science Core Collection, focusing on AI in blood glucose management. Data were analyzed using CiteSpace and VOSviewer to explore research trends, influential authors, and global collaborations.

**Results:**

The study revealed a substantial increase in publications, particularly after 2016. Major research clusters included CGM, machine learning algorithms, and predictive modeling. The United States, Italy, and the UK were prominent contributors, with key journals such as *Diabetes Technology & Therapeutics* leading the field.

**Conclusion:**

AI technologies are significantly advancing blood glucose management, especially through machine learning and predictive models. Future research should focus on clinical integration and improving accessibility to enhance patient outcomes.

## 1 Introduction

Diabetes mellitus (DM) represents a major global public health challenge. According to the International Diabetes Federation (IDF) Diabetes Atlas, the prevalence of diabetes is projected to reach 783 million by 2045, with 96% of these cases attributed to type 2 diabetes mellitus (T2DM) (1, 2). Despite significant financial and material investments by governments worldwide, efforts to prevent and manage diabetes have yielded suboptimal outcomes. Diabetes care still faces numerous challenges, including low awareness rates and poor glycemic control. For instance, in the United States, approximately one-quarter of the 33 million adult T2DM patients have poor glycemic control, with glycated hemoglobin (HbA1c) levels exceeding 8% (3). Lifestyle interventions are the cornerstone of diabetes treatment, but for individuals with poor glycemic control, pharmacological therapy is crucial. Achieving stable glycemic control through intensive insulin therapy often requires frequent dose adjustments and multiple blood glucose measurements, which pose significant challenges for most patients. Many patients visit clinics only every 3–6 months; however, due to therapeutic inertia, time constraints, and competing clinical demands, physicians frequently fail to optimize treatment regimens when needed (4). Consequently, glycemic control remains suboptimal for the majority of patients, highlighting the necessity of precision medicine to meet individual needs effectively.

In recent years, the rapid advancement of technology, including the emergence of platforms like ChatGPT, has led to the widespread application of artificial intelligence (AI). AI, a broad field, combines vast amounts of digital health data with the computational power needed to process these data (5). The rapid growth of digital medical data encompasses classic medical imaging (e.g., computed tomography [CT] scans, magnetic resonance imaging [MRI], nuclear medicine images) and digital pathology slides (e.g., endocrine tumor biopsies). Digital images also include real-world data, such as facial images for acromegaly detection, as well as images and videos captured during various medical and surgical procedures, such as retinal imaging for automated diabetic retinopathy screening. Textual data, including electronic health records (EHRs), large institutional databases, and health-related internet content like social media posts, are also expanding at an incredible pace. Additionally, “omics” fields contribute vast datasets for exploring individual characteristics, such as genomic sequencing for disease susceptibility or tumor diagnosis (5). Continuous health monitoring devices, ranging from approved medical tools like continuous glucose monitors (CGMs) to consumer devices like smartphones and smartwatches, further enhance data collection by tracking physical activity and dietary habits (5).

The introduction of AI has revolutionized endocrinology, providing powerful tools for the early diagnosis of diabetes and glycemic management (6–8). Wang et al. proposed a reinforcement learning (RL) framework, RL-DITR, based on a large-scale data model. This framework learns optimal insulin treatment strategies by analyzing glycemic state rewards from patient-model interactions. A single-group, patient-blinded trial involving 16 T2DM patients demonstrated that glycemic control was superior in the AI group compared to primary and mid-level physicians, without severe hypoglycemia or diabetic ketoacidosis (DKA) (9). A 2023 study published in JAMA found that, compared to participants receiving standard care, those in the AI-supported group achieved significantly better outcomes in time to optimal insulin dose, insulin adherence, glycemic control, and diabetes-related emotional distress (10). Similarly, Lee et al. reported that an AI-driven digital healthcare platform for T2DM improved glycemic levels and facilitated weight reduction (11).

Although T2DM accounts for the majority of diabetes cases, type 1 diabetes mellitus (T1DM) remains a critical concern. Due to the absolute deficiency of insulin, even with insulin pumps and CGMs, most T1DM patients fail to achieve glycemic targets (12). The ADVICE 4U trial, a 6-month multicenter, multinational, parallel, randomized non-inferiority study, included 108 T1DM patients aged 10–21 years. Participants were randomized to receive remote insulin dose adjustments every three weeks via an AI decision support system (AI-DSS) or physician guidance. Results indicated that AI-DSS was non-inferior to physician-led adjustments in time in range and insulin dose adjustments, with no diabetes-related adverse events reported in the AI-DSS group (compared to two cases of severe hypoglycemia and one DKA event in the physician group) (13). Rebecca et al. also found that AI-powered insulin dosage calculators for T1DM were both safe and effective (14).

Beyond glycemic control, AI has driven significant advancements in hardware development. Conventional insulin pumps combined with CGMs have improved glycemic outcomes and mitigated diabetes-related psychological burdens (15, 16). Subsequent innovations introduced low glucose suspend (LGS) and predictive LGS insulin pump therapies (17). Although these early systems reduced hypoglycemia risks, they did not fully address hyperglycemia (18). The current closed-loop systems mimic endogenous insulin release by delivering glucose-responsive insulin automatically. These systems are supported by three main algorithms: (1) model predictive control (MPC), which uses mathematical models of glucose regulation to determine optimal insulin infusion rates; (2) proportional-integral-derivative (PID) controllers, which adjust insulin delivery based on glucose deviations, trends, and rates of change; and (3) fuzzy logic algorithms that simulate clinical decision-making (19).

Despite the promising applications of AI in diabetes management, several challenges remain. Data privacy, especially in EHRs, poses a major concern. Additionally, the interpretability of AI models—how clinicians understand and trust AI-generated recommendations—remains a barrier. Furthermore, the scalability of AI solutions across diverse healthcare systems and cost-related constraints hinder widespread adoption. Addressing these challenges is critical to unlocking AI’s full potential in diabetes care.

This study aims to explore global research trends in AI-assisted blood glucose management through a bibliometric analysis. By examining key publications, collaborative networks, and emerging research themes, we seek to provide insights into the current state of AI applications in this field and to identify future opportunities and challenges.

## 2 Materials and methods

### 2.1 Methodology

Bibliometrics was first introduced in the early 1900s. It was established as an independent discipline in 1969 by Pritchard (20) and later became widely applied in literature analysis (21). This analytical method provides a quantitative approach to review and investigate the extant literature within a given field (22). It captures details such as authors, keywords, journals, countries, institutions, and references, which can then be analyzed. The development of this field has been bolstered by bibliometric analysis (23). With modern computer technology and graphical visualization tools, bibliometric analysis has become an essential supplement to traditional literature reviews.

Co-citation analysis is another frequently employed method within bibliometrics. It is defined as the relationship between two articles cited by one or more other articles simultaneously. Ma and Xi emphasized that visualized co-citation analysis facilitates data interpretation, making results more comprehensive. This method can be applied to a variety of items, including authors, keywords, institutions, and countries, which enhances the overall analysis of the selected papers. The visualization provides insight into the internal relationships between information, such as shared research topics, emergent theories, or connections between established and novel fields.

Data visualization and trend analysis were performed using CiteSpace and VOSviewer. While these tools have been validated in numerous bibliometric studies, we acknowledge that their data granularity and visualization accuracy may vary. To address potential outliers or anomalies in the dataset, statistical methods (e.g., interquartile range analysis and sensitivity testing) were applied, and any extreme values were examined to ensure they did not unduly influence the overall trends.

### 2.2 Data collection

On January 7, 2025, the two authors independently conducted a comprehensive search using the Web of Science Core Collection database. The search was designed to identify articles related to “AI-assisted blood glucose management” by including a range of relevant keywords and phrases. These keywords included, but were not limited to, the following: (“artificial intelligence” OR “AI” OR “machine learning” OR “deep learning” OR “neural network” OR “algorithm”) AND (“blood glucose management” OR “blood sugar control” OR “glycemic control” OR “glucose monitoring”) AND (“diabetes” OR “type 1 diabetes” OR “type 2 diabetes”) AND (“continuous glucose monitoring” OR “CGM” OR “insulin adjustment” OR “prediction models”). The search period spanned from January 1, 2006, to January 1, 2025, and was limited to English-language publications. Non-peer-reviewed sources such as news reports, conference abstracts, editorials, and general health science disseminations were excluded to ensure a focus on established research findings. Salient data, including titles, keywords, author information, abstracts, citations, and publication details, were retrieved and exported in TXT format for further analysis.

**Figure 1:**
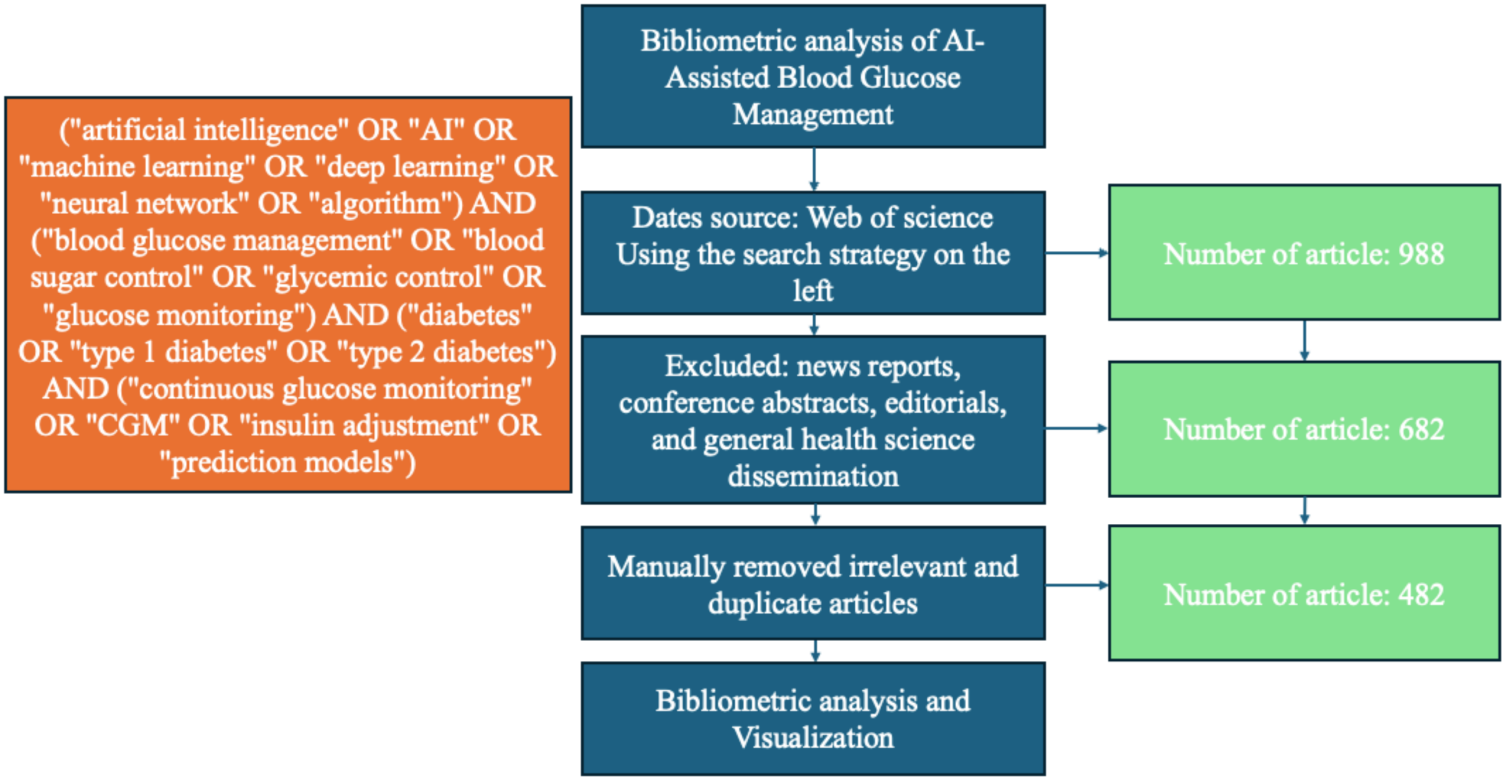
Flowchart of bibliometric analysis.

Subsequently, the two authors reviewed the collected articles independently to remove those that were not pertinent to the core theme of AI-assisted blood glucose management. After this refinement process, a total of 482 references were deemed relevant and included for bibliometric analysis (**Figure 1**).

Our analysis is based solely on the Web of Science Core Collection. Although this database is recognized for its rigorous indexing, we recognize that excluding other databases (such as Scopus or PubMed) and non-English sources may omit some relevant studies.

We deliberately excluded non-peer-reviewed sources to focus on studies that have undergone rigorous evaluation. While this may omit some early-stage or innovative findings, we believe that it enhances the reliability of the trends identified in our bibliometric analysis.

### 2.3 Statistical methods

We used CiteSpace and VOSviewer, two visualization tools, for this analysis. The versions employed in this study were CiteSpace 6.3.1 and VOSviewer 1.6.20. CiteSpace was utilized to generate Timezone and Timeline visualizations, clearly illustrating the evolutionary trajectory of knowledge within the field of AI-assisted blood glucose management. These visualizations helped identify key research trends and significant contributions over time (24). For example, the Timezone map highlighted emerging topics such as machine learning algorithms for continuous glucose monitoring.

In contrast, VOSviewer provided Network Visualization, Overlay Visualization, and Density Visualization, offering a comprehensive view of co-occurrence patterns among keywords, authors, and institutions. Its user-friendly interface and visually appealing outputs enabled clearer identification of research clusters and collaborative networks (25). By employing both tools, we were able to uncover prominent contributors, key research domains, and evolving trends, providing a comprehensive understanding of the field’s development.

## 3 Results and analysis

This study included 482 papers authored by 2,396 researchers from 883 institutions across 61 countries. These papers were published in 172 journals and cited 13,928 references from 4,105 different journals.

### 3.1 Analysis of the source of the article’s publication

#### 3.1.1 Annual growth trend of publications

**Figure 2:**
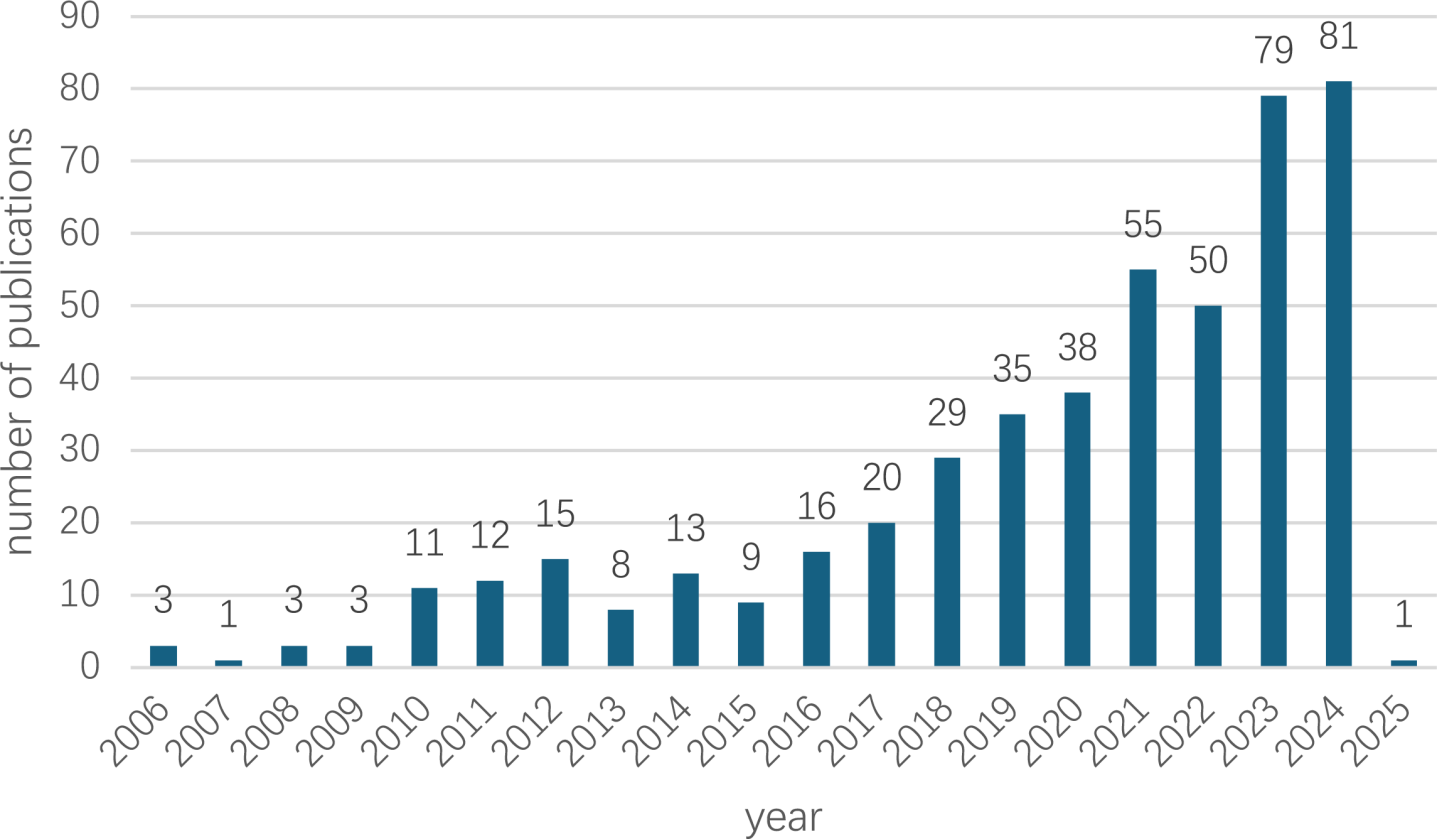
Annual growth trend of publications. The annual publication trend of AI-assisted blood glucose management research from 2006 to 2025. The data for 2025 is based on publications retrieved up to January 7, 2025.

The bar chart offers a comprehensive view of the annual number of publications on AI-assisted blood glucose management from 2006 to 2025 (**Figure 2**). During the initial period from 2006 to 2010, publication numbers remained consistently low, averaging fewer than four papers per year, reflecting limited research activity in this domain at the time.

From 2011 to 2015, a gradual upward trend emerged, with annual publications ranging from 8 to 15, signaling an increasing interest in the potential of AI technologies for diabetes management. This upward trajectory gained significant momentum from 2016 onward, as evidenced by a steady increase in publications, reaching 29 in 2018 and 55 in 2022.

The most remarkable growth occurred between 2023 and 2024, with the number of publications peaking at 81 in 2024, underscoring the growing global focus on this research field. For 2025, only one publication has been recorded, as the dataset was extracted on January 7, 2025, indicating that the final count for the year remains incomplete.

Overall, the data reveals a sharp and sustained upward trend, particularly over the past decade, coinciding with rapid advancements in AI technologies and their expanding applications in healthcare.

#### 3.1.2 High-impact authors and collaborative networks

**Figure 3:**
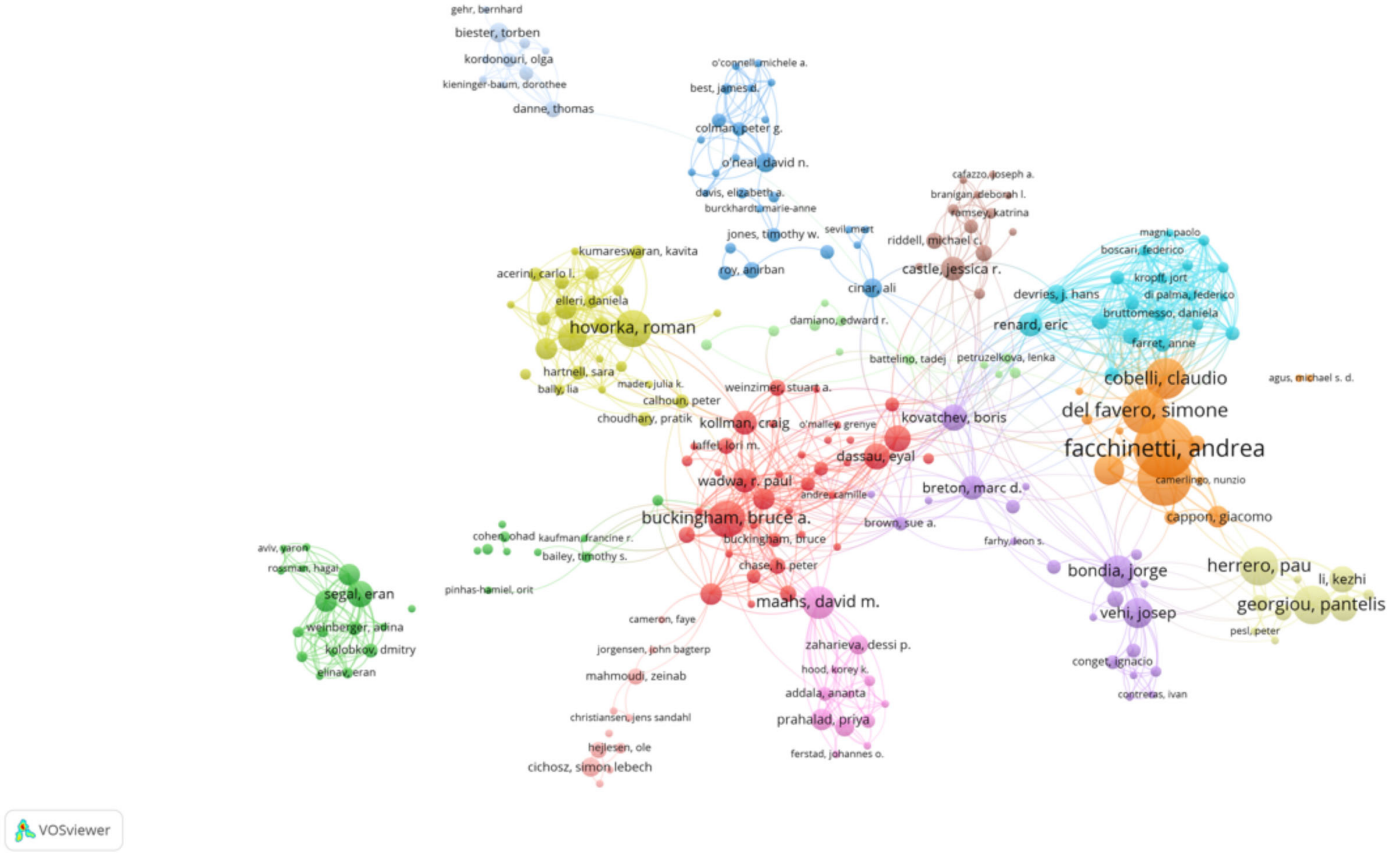
High-productivity authors’ collaboration network. Visualization of collaboration networks among authors with at least two publications. Larger nodes represent authors with higher publication counts, while edges indicate co-authorship relationships.

The network visualization map above depicts the collaborative relationships among authors who have published at least two papers on AI-assisted blood glucose management. Each node represents an author, and the size of the node corresponds to the number of publications attributed to that author. The connections between nodes indicate co-authorship, with thicker lines representing stronger collaborative ties (**Figure 3**).

Several prominent clusters are visible, each representing a distinct group of closely collaborating authors. The red cluster, for example, highlights influential authors such as Bruce Buckingham and David M. Maahs, who are known for their contributions to continuous glucose monitoring and diabetes management. Similarly, the yellow cluster centers around Roman Hovorka, a key figure in developing closed-loop insulin delivery systems.

Other significant clusters include:

- The blue cluster, led by Claudio Cobelli and Andrea Facchinetti, who focus on mathematical modeling and algorithm development.
- The green cluster, featuring authors such as Pantelis Georgiou, who work on sensor technologies and artificial intelligence applications in diabetes care.

The map highlights the interdisciplinary and collaborative nature of research in this field, with strong connections between experts in endocrinology, biomedical engineering, and computer science. This network underscores the importance of teamwork in advancing AI technologies for diabetes management.

#### 3.1.3 High-productivity authors

High-productivity authors have significantly advanced the field of AI-assisted blood glucose management. Facchinetti A leads with 27 publications and 641 citations, followed by Sparacino G with 23 publications and 562 citations (**Table 1**). Both have contributed extensively to algorithm development and continuous glucose monitoring. Del FS and Cobelli C have fewer publications but boast high citation averages of 39.11 and 51.63, respectively, highlighting the impact of their work on insulin delivery technologies and physiological modeling. Georgiou P, with 15 publications, specializes in sensor development and AI integration, further enriching this domain. These authors represent the forefront of research, driving technological innovations and improving clinical outcomes.

**Table 1:** High-productivity authors in AI-assisted blood glucose management research.

Rank	Author	Documents	Citations	Average citation/Publication
1	Facchinetti A	27	641	23.74
2	Sparacino G	23	562	24.43
3	Del FS	18	704	39.11
4	Cobelli C	16	826	51.63
5	Georgiou P	15	466	31.06

This table lists the top five authors ranked by their total number of publications, citations, and average citations per publication.

#### 3.1.4 High-impact journals

The top 10 high-impact journals in AI-assisted blood glucose management demonstrate the field’s interdisciplinary nature and research quality. “Diabetes Technology & Therapeutics” leads with 75 publications and 2,505 citations, emphasizing innovations in continuous glucose monitoring and insulin delivery (**Table 2**). “Sensors,” with 26 publications, focuses on sensor technology, while “Diabetes Care,” although ranking third in publication count, has the highest average citation per publication (66.7), reflecting its role as a premier source for clinical diabetes research. Engineering-focused journals, such as “IEEE Journal of Biomedical and Health Informatics” and “IEEE Transactions on Biomedical Engineering,” also feature prominently, with 15 publications each, bridging AI and biomedical applications. “Pediatric Diabetes” highlights research tailored to younger populations, while journals like “Diabetes Obesity & Metabolism” and “Scientific Reports” underscore the breadth of research spanning clinical, technical, and basic science. This distribution underscores the pivotal role of these journals in disseminating impactful research and advancing the integration of AI technologies in diabetes care.

**Table 2:** High-impact journals in AI-assisted blood glucose management research.

Rank	Source	Publication	Citation	Average citation/Publication
1	diabetes technology & therapeutics	75	2505	33.4
2	sensors	26	720	27.7
3	diabetes care	22	1467	66.7
4	IEEE journal of biomedical and health informatics	15	399	26.6
5	IEEE transactions on biomedical engineering	15	502	33.5
6	pediatric diabetes	12	291	24.3
7	diabetes obesity & metabolism	11	240	21.8
8	computer methods and programs in biomedicine	10	186	18.6
9	frontiers in endocrinology	10	42	4.2
10	scientific reports	10	112	11.2

This table lists the top 10 journals ranked by publication count, citations, and average citations per publication.

#### 3.1.5 National contributions to research

The two figures above highlight the global distribution of research contributions in AI-assisted blood glucose management. The first table ranks the top five countries by publication count, citations, and average citations per publication, while the second figure presents a collaborative network map of countries actively involved in this field (**Table 3**).

**Table 3:** Top five countries in AI-assisted blood glucose management research.

Rank	Source	Publication	Citation	Average citation/Publication
1	USA	155	5607	36.17
2	Italy	67	2136	31.88
3	England	56	2115	37.76
4	China	44	438	9.95
5	Spain	39	829	21.25

This table lists the top five countries ranked by publication count, total citations, and average citations per publication.

The United States leads the field with 155 publications and 5,607 citations, showcasing its dominant position in terms of research output and influence. Its average of 36.17 citations per publication reflects the high quality and impact of its research. Following the U.S., Italy ranks second with 67 publications and 2,136 citations, highlighting its significant contributions, particularly in clinical applications and technological advancements. England takes third place with 56 publications and a slightly higher average citation per publication (37.76), reflecting the strong influence of its academic and medical research institutions. China and Spain round out the top five, with 44 and 39 publications, respectively, demonstrating their active engagement in collaborative research and technology development.

**Figure 4:**
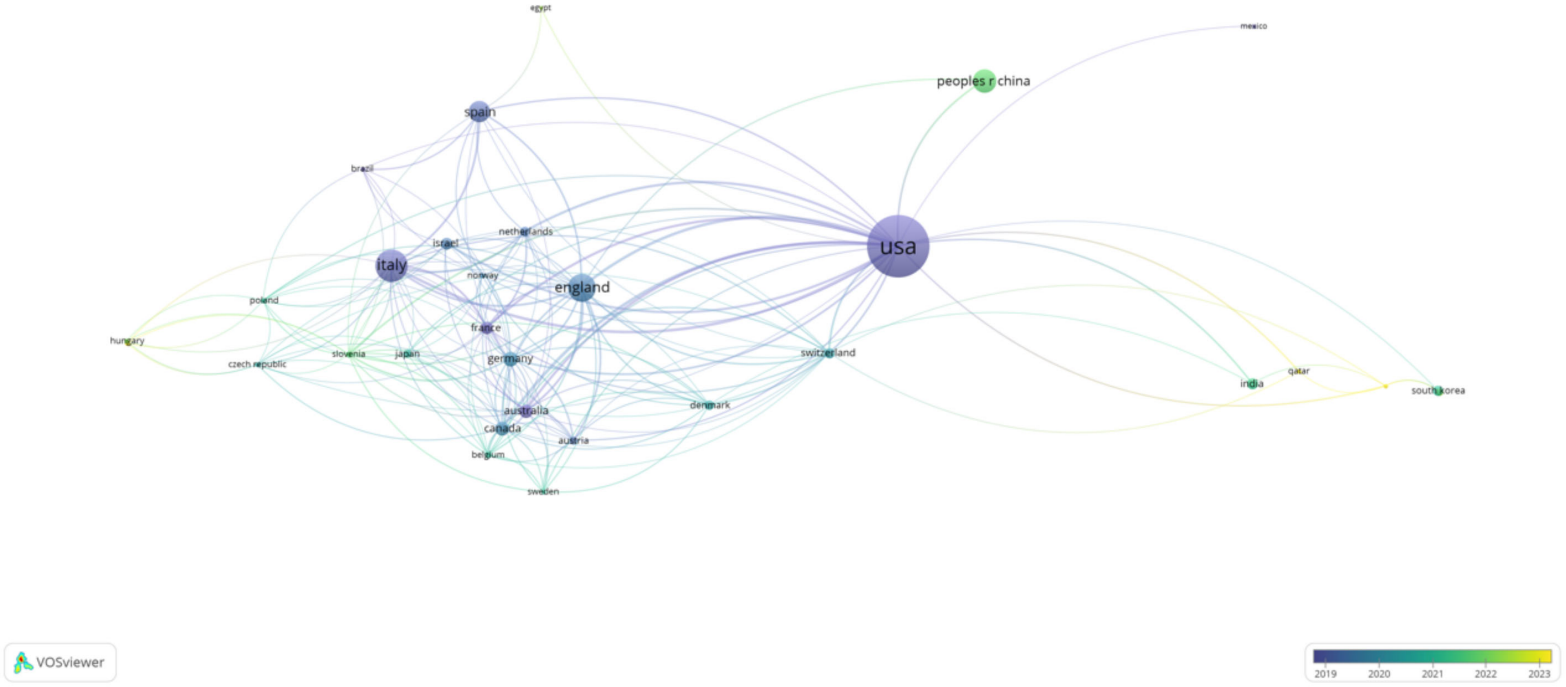
International Collaboration Network by Country. This network map illustrates collaborations between countries actively contributing to AI-assisted blood glucose management research, generated using VOSviewer.

The second figure visually illustrates the international collaboration network, with the United States acting as the central hub. Strong connections are observed between the U.S. and major research partners such as Italy, England, and Germany, reflecting frequent collaborative efforts. Emerging contributions from countries like China and South Korea indicate the growing global interest in this research area, with increasing participation from Asian institutions (**Figure 4**). The visualization underscores the importance of international partnerships in advancing the field, facilitating knowledge exchange, and fostering innovative solutions to improve diabetes management.

These findings highlight the geographical diversity of research contributions, with developed nations leading the charge and developing regions gradually increasing their participation, which is crucial for addressing global healthcare challenges.

#### 3.1.6 Keyword co-occurrence and research hotspots

**Figure 5:**
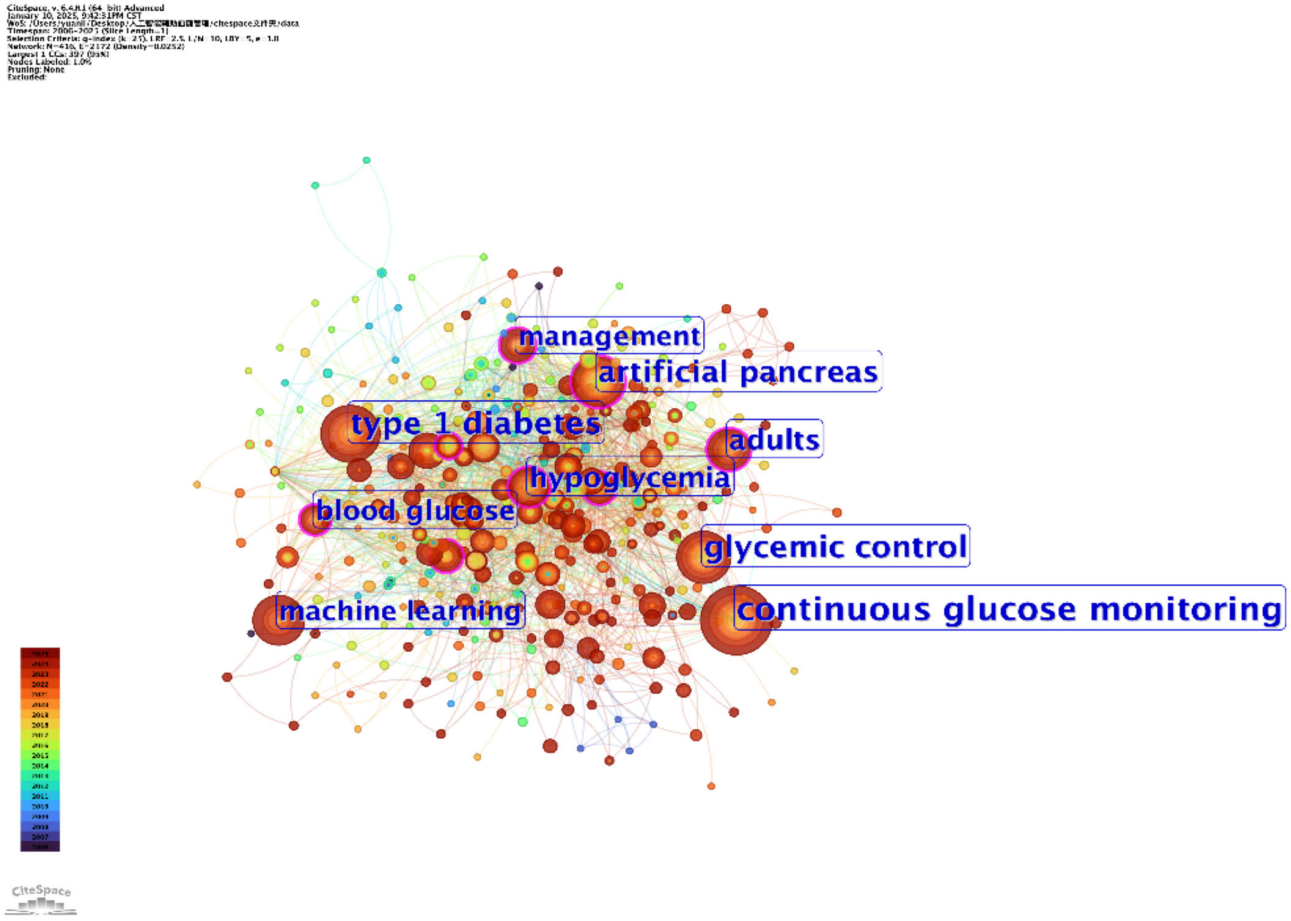
Keyword co-occurrence map (CiteSpace). This map, generated using CiteSpace, visualizes the relationships between high-frequency keywords in AI-assisted blood glucose management research.

**Figure 6:**
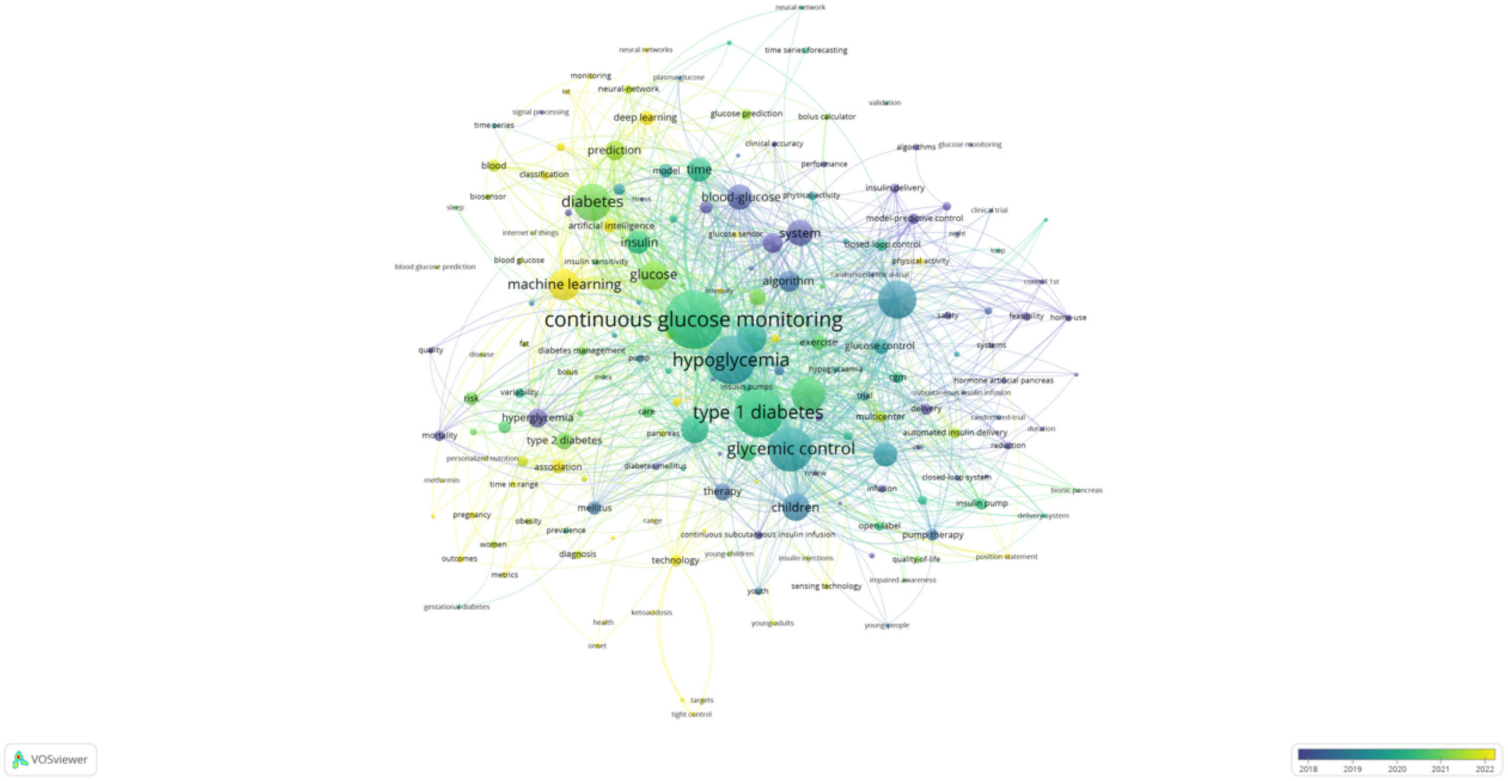
Keyword co-occurrence map (VOSviewer). This map, generated using VOSviewer, displays the co-occurrence relationships among keywords in AI-assisted blood glucose management research.

The two keyword co-occurrence maps, generated using CiteSpace (**Figure 5**) and VOSviewer (**Figure 6**), reveal the key research hotspots in AI-assisted blood glucose management. These maps visually represent the relationships between high-frequency keywords, illustrating the central themes and emerging trends in the field.

The CiteSpace map identifies “continuous glucose monitoring,” “glycemic control,” and “type 1 diabetes” as core keywords, reflecting their central role in current research. Keywords such as “machine learning” and “artificial pancreas” highlight the increasing integration of AI technologies into clinical applications, particularly in the development of advanced insulin delivery systems and real-time glucose monitoring solutions. Additionally, the temporal aspect of the CiteSpace map shows how these research areas have evolved over time, with recent emphasis on predictive modeling and personalized treatment strategies.

The VOSviewer map complements these findings by providing a detailed network of keyword relationships. The size of each node represents the frequency of the keyword, while the proximity between nodes indicates their co-occurrence strength. “Continuous glucose monitoring” is prominently featured as the most interconnected keyword, linking to terms like “hypoglycemia,” “glycemic control,” and “machine learning.” This underscores the importance of CGM technology in driving advancements in diabetes care. Furthermore, emerging keywords such as “prediction” and “children” reflect growing interest in predictive analytics and pediatric diabetes management.

Together, these keyword co-occurrence analyses highlight the diverse research focus within the field, from technological innovations like AI-driven glucose sensors to clinical applications aimed at improving patient outcomes. The visualizations provide valuable insights into the research priorities and collaborative opportunities that will shape the future of AI-assisted diabetes management.

#### 3.1.7 Keyword clustering analysis

**Figure 7:**
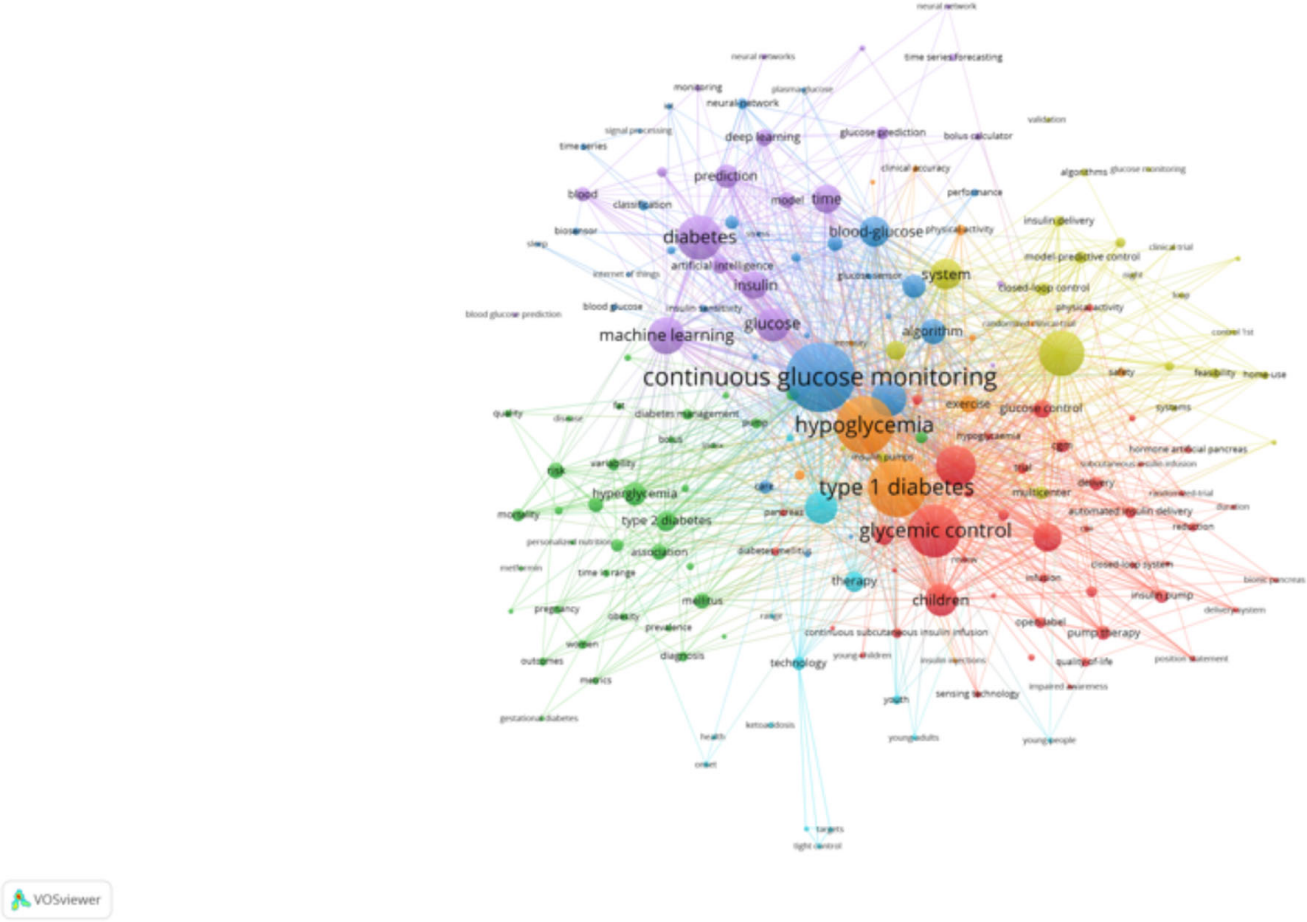
Keyword clustering map (VOSviewer). This map, generated using VOSviewer, shows clusters of keywords representing distinct research themes in AI-assisted blood glucose management.

**Figure 8:**
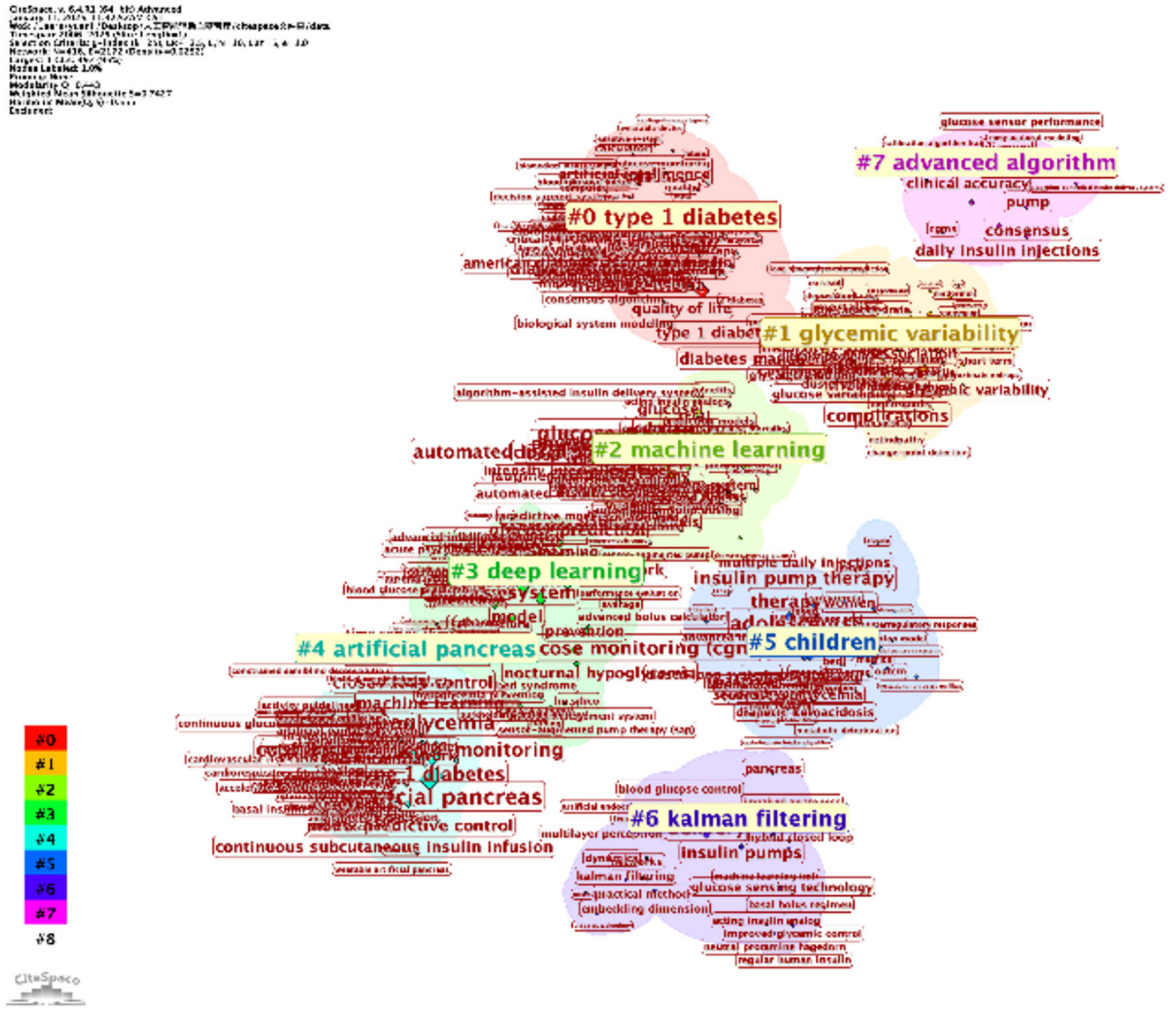
Keyword clustering map (CiteSpace). This map, generated using CiteSpace, illustrates clusters of keywords representing major research themes in AI-assisted blood glucose management.

The keyword clustering analyses, generated using VOSviewer (**Figure 7**) and CiteSpace (**Figure 8**), reveal distinct research themes within AI-assisted blood glucose management.

The VOSviewer map shows several key clusters. The red cluster focuses on “continuous glucose monitoring,” “type 1 diabetes,” and “glycemic control,” highlighting the central role of monitoring and control technologies. The green cluster emphasizes “machine learning” and “prediction,” reflecting the integration of AI for predictive models in diabetes care. Meanwhile, the blue and yellow clusters address clinical aspects, such as “hypoglycemia” and “artificial pancreas,” showcasing the application of automation in treatment. These clusters indicate the convergence of clinical, technological, and algorithmic research.

The CiteSpace map further refines this analysis with seven major clusters, including “type 1 diabetes” (Cluster #0) and “machine learning” (Cluster #2). Notably, “glycemic variability” (Cluster #1) and “artificial pancreas” (Cluster #4) reflect specialized research directions, while newer clusters, such as “deep learning” (Cluster #3) and “advanced algorithms” (Cluster #7), indicate emerging trends. The temporal visualization also reveals the evolution of research, with recent years focusing heavily on automation and AI-driven decision-making.

These clustering results provide a clear picture of the field’s current focus and its progression, offering valuable insights for future research directions.

#### 3.1.8 Burst terms and emerging trends

**Figure 9:**
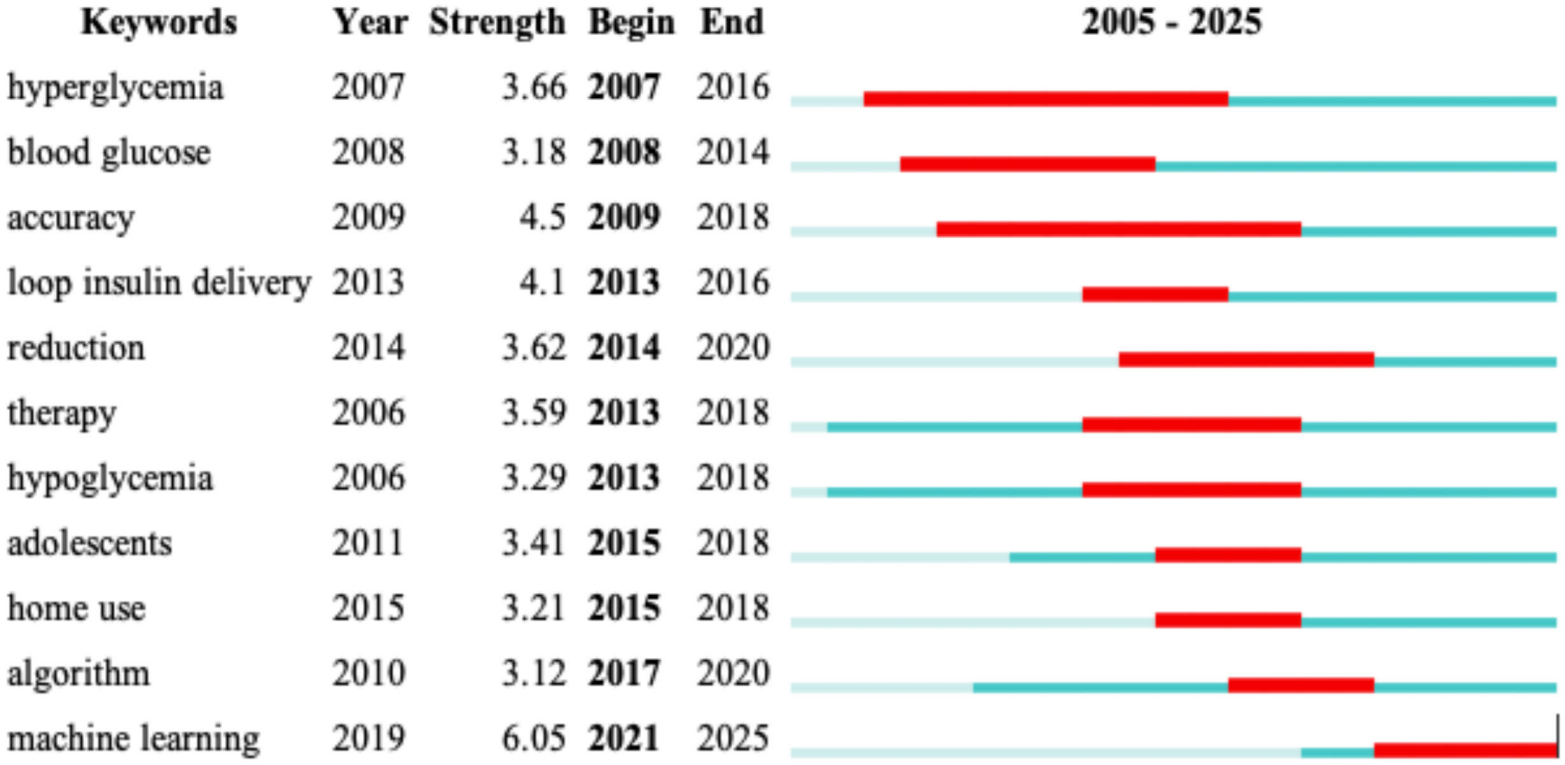
Burst terms in AI-assisted blood glucose management. This table visualizes keywords that experienced a sudden surge in research attention over specific periods, generated using CiteSpace.

The figure above highlights burst terms, indicating keywords that experienced a sudden increase in research attention over specific time periods (**Figure 9**). These terms provide valuable insights into emerging trends and shifts in research focus within AI-assisted blood glucose management.

Key burst terms include “hyperglycemia” (2007–2016) and “blood glucose” (2008–2014), which reflect foundational topics in diabetes research during earlier years. “Accuracy” (2009–2018) and “loop insulin delivery” (2013–2016) illustrate a growing focus on improving the precision of glucose monitoring and the development of automated insulin delivery systems. Notably, “reduction” (2014–2020) indicates a continued interest in strategies to reduce complications associated with diabetes.

In more recent years, keywords such as “algorithm” (2017–2020) and “machine learning” (2021–2025) have emerged as major research foci, reflecting the rapid integration of advanced AI technologies. “Home use” and “adolescents” (2015–2018) highlight efforts to broaden the accessibility and applicability of diabetes management tools, particularly for younger populations.

These burst terms underscore the dynamic nature of this research field, showcasing both foundational themes and cutting-edge innovations that are shaping the future of diabetes care.

#### 3.1.9 Timeline and temporal analysis

The timeline and temporal analysis, presented in two visualizations, illustrate the evolution of research topics and their interconnections over time.

**Figure 10:**
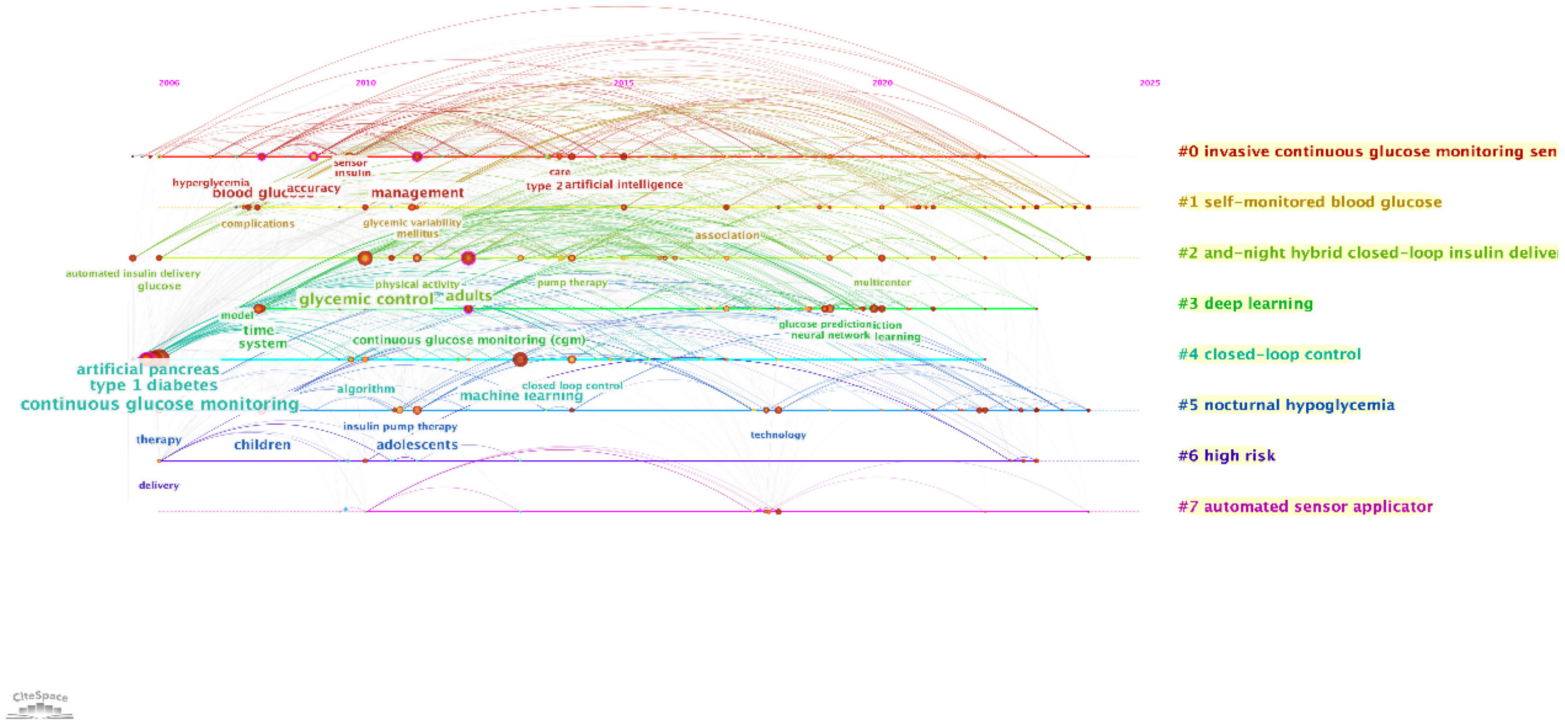
Timeline visualization of research topics. This timeline, generated using CiteSpace, presents the evolution of major research clusters in AI-assisted blood glucose management over time.

The first timeline map, generated using CiteSpace, shows the progression of major research themes from 2005 to 2025 (**Figure 10**). Prominent topics such as “continuous glucose monitoring,” “type 1 diabetes,” and “glycemic control” dominate the early years, reflecting foundational work in diabetes management technologies. Over time, emerging topics like “artificial intelligence,” “machine learning,” and “deep learning” gain prominence, signaling a shift towards integrating AI in predictive modeling and personalized treatment strategies. The sequential connections between nodes indicate the flow of research focus, while the color coding highlights the temporal emergence of each theme.

**Figure 11:**
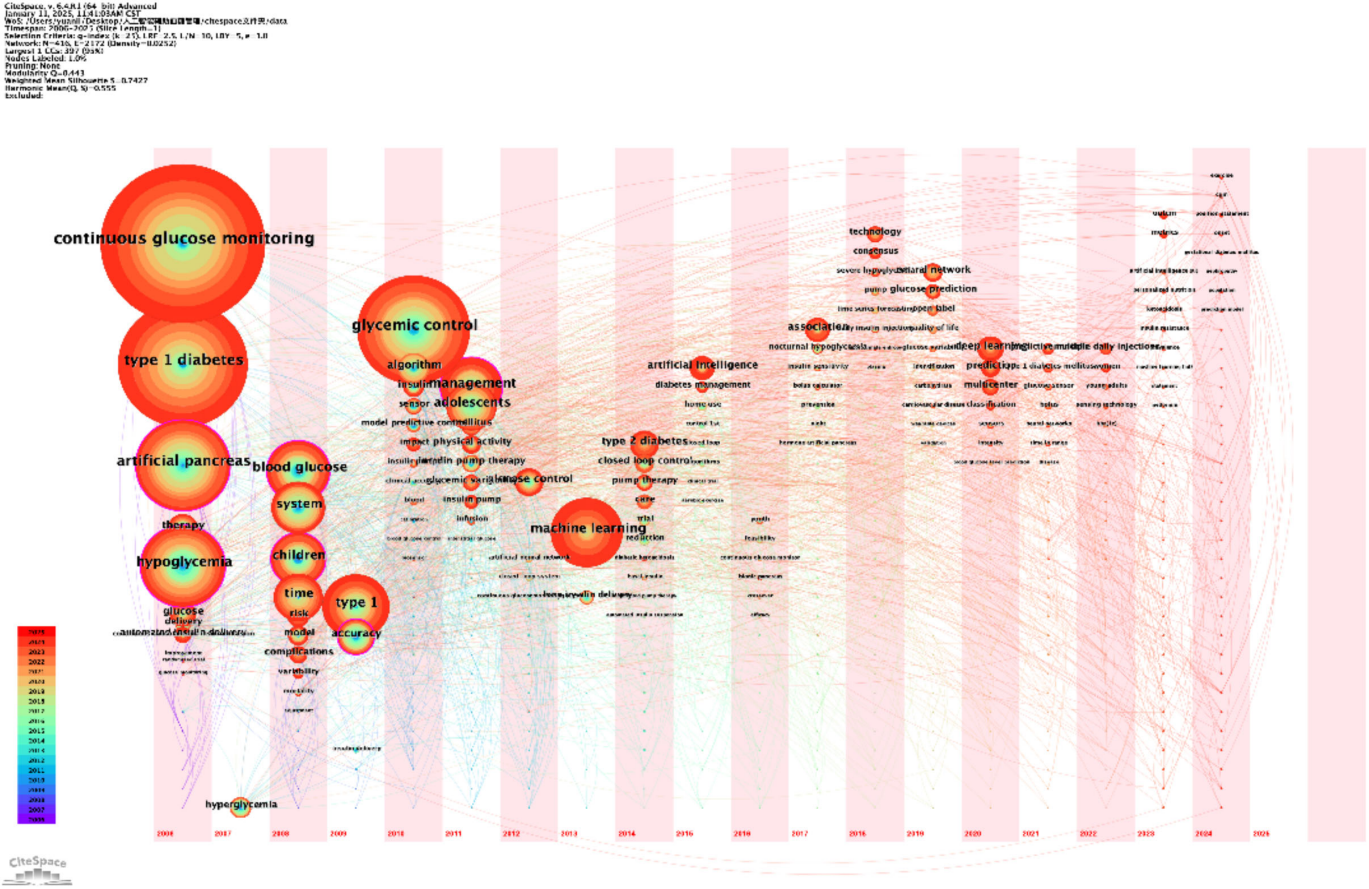
Temporal visualization of research themes. This temporal visualization, generated using CiteSpace, showcases the progression and development of key research themes in AI-assisted blood glucose management over time.

The second temporal map, also from CiteSpace, emphasizes the clustering of research topics and their temporal relationships (**Figure 11**). Clusters like “closed-loop control” and “automated sensor applicator” illustrate recent advancements in automation and real-time glucose management. Additionally, topics such as “nocturnal hypoglycemia” and “self-monitored blood glucose” highlight ongoing challenges and areas for innovation in improving patient safety and convenience.

These analyses provide a clear temporal framework for understanding the development of AI-assisted blood glucose management. They reveal how foundational research has paved the way for innovative applications, offering a roadmap for future investigations in this rapidly evolving field.

#### 3.1.10 Research hotspots visualization

**Figure 12:**
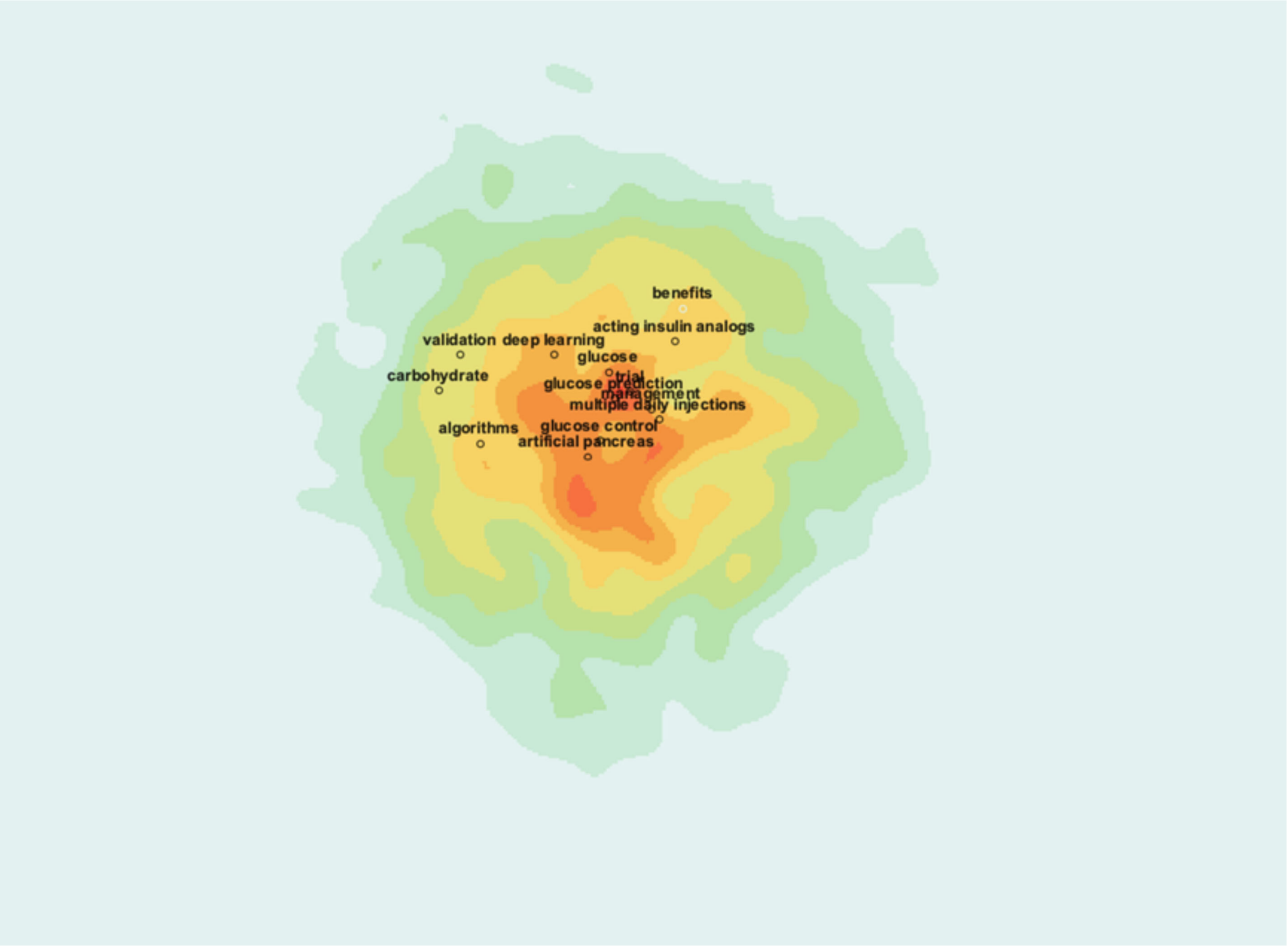
Research hotspot heatmap.

The heatmap visualization above highlights the core research hotspots in AI-assisted blood glucose management, with colors indicating the intensity of research activity (**Figure 12**). The central red zone represents areas of high research concentration, with key terms such as “artificial pancreas,” “glucose control,” and “algorithms” prominently featured. These terms signify the field’s focus on technological innovations aimed at automating glucose management and enhancing treatment precision.

Surrounding this core are additional hotspots, including “deep learning,” “carbohydrate,” and “multiple daily injections,” which indicate specific subfields such as advanced computational methods and practical treatment strategies. The outer green and yellow zones show emerging or less-studied areas, such as “validation” and “acting insulin analogs,” suggesting directions for future research.

This visualization provides a clear and intuitive understanding of the field’s primary focus areas, emphasizing the importance of integrating AI with clinical practices to optimize diabetes management.

#### 3.1.11 Co-citation analysis of journals and authors

**Figure 13:**
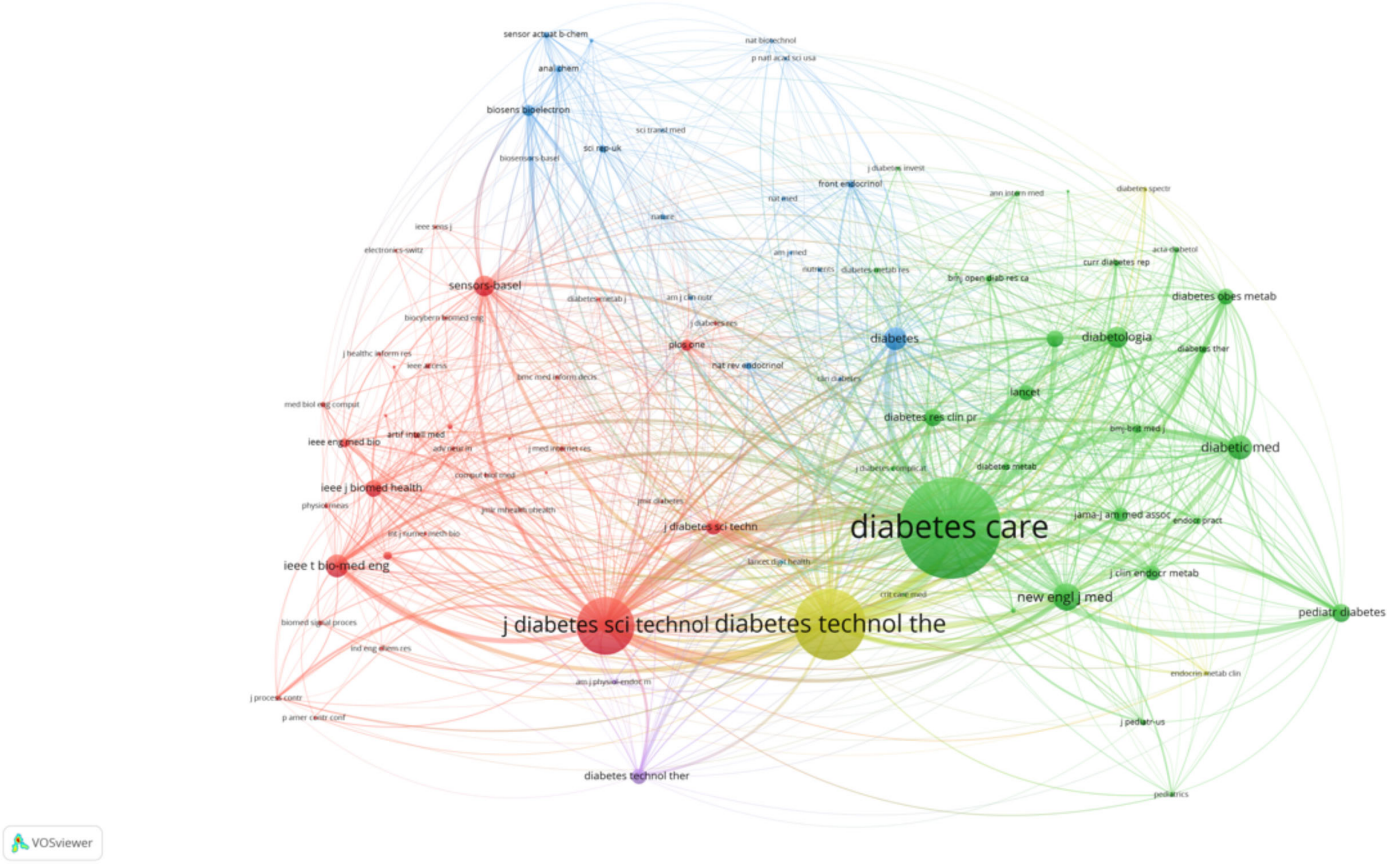
Journal co-citation analysis.

The first co-citation map highlights the key journals that have significantly influenced AI-assisted blood glucose management (**Figure 13**). “Diabetes Care” emerges as the most prominent journal, signifying its central role in disseminating high-impact research. Closely following are journals such as “Diabetes Technology & Therapeutics” and “Pediatric Diabetes,” which emphasize technological advancements and specialized care for pediatric populations, respectively. Other journals, including “IEEE Transactions on Biomedical Engineering” and “Journal of Diabetes Science and Technology,” showcase the integration of engineering and clinical research, further emphasizing the interdisciplinary nature of this field.

**Figure 14:**
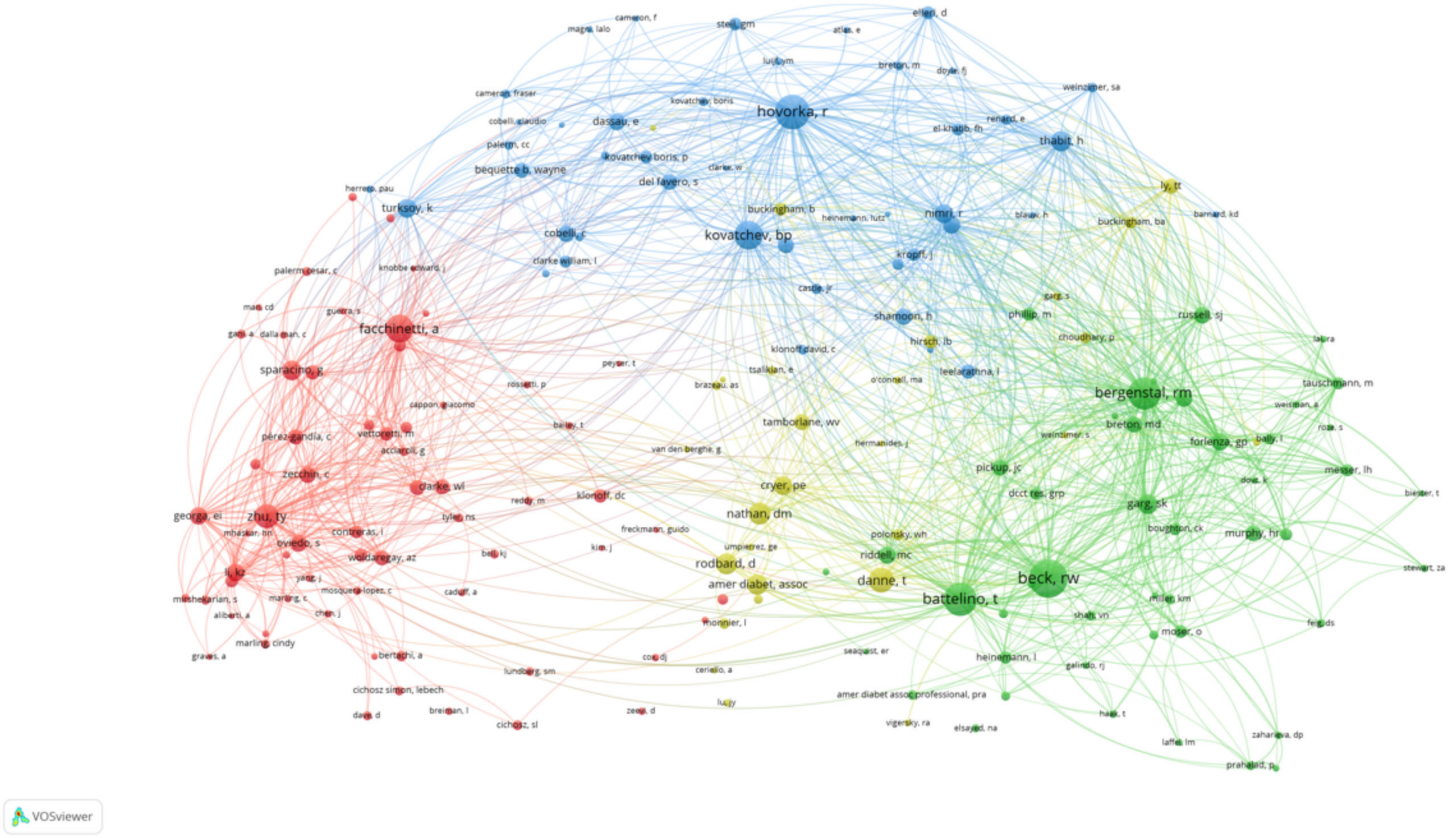
Author co-citation analysis.

The second co-citation map focuses on influential authors (**Figure 14**). Key figures such as Roman Hovorka and Bruce Buckingham stand out, reflecting their substantial contributions to closed-loop systems and continuous glucose monitoring. David M. Maahs and Claudio Cobelli also appear prominently, underlining their work in physiological modeling and technological innovation. These authors are interconnected through collaborative networks, indicating a shared commitment to advancing AI-driven diabetes care. This co-citation analysis underscores the foundational role of both journals and authors in shaping the research landscape and driving progress in AI-assisted diabetes management.

### 3.2 Discussion

This study employed bibliometric analysis to provide a comprehensive overview of the research landscape in AI-assisted blood glucose management over nearly two decades. By integrating multiple visualization tools such as CiteSpace (24) and VOSviewer (25), we mapped the evolution of research topics, collaborative networks, and emerging trends, thereby offering valuable insights into the field’s development in the context of the global diabetes burden (1, 2).

#### 3.2.1 Summary of key findings

Our analysis revealed a sustained upward trend in publications since 2006, with a marked acceleration over the past decade. This trend is consistent with rapid advancements in AI technologies (5–8) and their increasing application in healthcare, particularly in diabetes management (9, 10). Notably, the surge in publication numbers observed between 2023 and 2024 reflects both the growing global interest in AI‐driven approaches and the emergence of subfields such as predictive analytics and automated insulin delivery (11, 12). These trends are further supported by recent clinical studies demonstrating improved glycemic control using AI‐based interventions (13–15).

The collaborative network analysis highlighted the interdisciplinary nature of research in this field. Prominent authors from clinical, engineering, and computer science backgrounds have formed robust collaborative clusters, underscoring the importance of cross‐disciplinary efforts in addressing the complex challenges of diabetes care (16, 17). In addition, the geographical distribution of research contributions indicates that while developed countries dominate publication output, emerging contributions from Asian institutions signal an increasingly global effort in integrating AI into clinical practice (18, 19).

Keyword co‐occurrence and clustering analyses identified several core themes, including continuous glucose monitoring, machine learning, and the development of artificial pancreas systems. These themes not only reflect the current state of research but also highlight emerging areas such as deep learning‐based predictive models and personalized treatment strategies (21, 23). Moreover, burst term analysis captured dynamic shifts in research focus, with recent years emphasizing real‐time decision‐making and home‐based applications of AI in diabetes management (22, 26).

#### 3.2.2 Interpretation and implications

The observed steady increase in research output and the emergence of prominent collaborative clusters suggest that the integration of AI in blood glucose management is transitioning from conceptual exploration to widespread clinical application (27, 28). The centrality of continuous glucose monitoring and glycemic control in the keyword maps reinforces the clinical relevance of these technological innovations (29, 30). Furthermore, the rising prominence of machine learning and deep learning in the literature highlights an ongoing evolution toward more sophisticated, data‐driven decision‐making systems (31, 32).

The interdisciplinary and international collaborative patterns observed underscore the necessity of combining diverse expertise to address the multifaceted nature of diabetes management (33–35). For instance, recent studies employing reinforcement learning have demonstrated promising results in optimizing glycemic control (36), while digital health platforms incorporating AI‐based dietary management have improved patient outcomes in type 2 diabetes (9). These findings have practical implications: clinicians can benefit from advanced AI tools for precise glycemic management, and engineers are encouraged to develop robust algorithms that can accommodate the variability inherent in clinical data (37, 38).

#### 3.2.3 Comparison with previous literature

Our findings align with prior bibliometric studies documenting the increasing impact of AI in healthcare research (33, 34). However, this study specifically emphasizes the rapid evolution of AI applications in diabetes care—a trend that mirrors developments in other medical fields such as oncology and cardiovascular medicine (1, 2). The significant increase in research output after 2016 parallels the broader adoption of machine learning methods in clinical studies (5, 8), reinforcing the notion that technological breakthroughs can accelerate both academic and clinical advancements (13, 15).

#### 3.2.4 Citation count bias and its limitations

Although citation counts are commonly employed as a quantitative metric to assess the impact of research publications, they possess inherent limitations. For instance, older publications tend to accumulate more citations due to their longer exposure, which may bias the analysis against more recent, yet potentially high-quality studies. Furthermore, citation practices can be influenced by various factors, such as self-citation, journal reputation, and disciplinary differences, which may not directly reflect the clinical relevance or methodological rigor of the research. In our study, while citation counts provided a useful overview of the evolution of research trends in AI-assisted blood glucose management, we acknowledge that relying solely on this metric may oversimplify the nuanced influence of individual studies. Future analyses could benefit from incorporating additional metrics—such as expert qualitative assessments—to offer a more balanced evaluation of research impact.

#### 3.2.5 Linking bibliometric trends to clinical outcomes

Our revised Discussion section now more explicitly connects the bibliometric trends identified with real-world clinical outcomes. Specifically, we have integrated the following changes:

• Translation of AI Technologies into Clinical Practice:

We now discuss that the increasing focus on continuous glucose monitoring, machine learning algorithms, and predictive models—as revealed by our bibliometric analysis—corresponds with their emerging clinical applications. For example, studies by Nimri (13) and Unsworth (14) have demonstrated that AI-based decision support systems can optimize insulin dosing, resulting in improved glycemic control and reduced hypoglycemic events.

• Impact of Predictive Models on Patient Outcomes:

We elaborate on how predictive models, which form a major research cluster in our analysis, are being validated and integrated into clinical practice. Research by Thomas (15) and Beck (16) illustrates that these models not only enhance individualized insulin therapy but also have the potential to improve long-term patient outcomes, such as reducing diabetes-related complications.

• Need for Further Clinical Validation:

While bibliometric trends show growing interest in AI applications, we now emphasize that many studies remain in preliminary stages. We call for future research that combines bibliometric data with clinical outcome assessments to rigorously evaluate the efficacy of these AI tools in routine practice (e.g., linking improvements in glycemic variability with AI-driven interventions).

#### 3.2.6 Implications for future clinical practice and healthcare equity

Emerging AI-driven technologies in blood glucose management not only promise to improve clinical outcomes but also offer significant potential for enhancing healthcare equity (11, 16). The integration of continuous glucose monitoring, predictive models, and telemedicine platforms can extend high-quality diabetes care to underserved and remote populations, thereby reducing disparities in access and outcomes (39, 40). These digital interventions, by enabling more personalized and timely treatment adjustments, may help ensure that advances in diabetes management benefit a broader, more diverse patient population, ultimately fostering a more inclusive healthcare system.

#### 3.2.7 Limitations of co-citation analysis

It is important to acknowledge that while co-citation analysis is effective in mapping collaborative networks and identifying research clusters, it does not directly assess the quality or clinical impact of the cited works. Although this method provides a valuable overview of the intellectual structure within the field, it may overlook nuances such as study design rigor and direct clinical relevance. Thus, incorporating additional qualitative analyses would be essential for a more comprehensive evaluation of research influence.

#### 3.2.8 Detailed analysis of predictive models

Our analysis identified predictive models as a major research cluster within the field of AI-assisted blood glucose management. Although our bibliometric study primarily focused on mapping research trends rather than evaluating algorithmic performance, several key studies have validated predictive algorithms with promising results. For instance, Nimri (13) demonstrated that AI-based decision support systems can optimize insulin dosing by accurately predicting blood glucose fluctuations in youths with type 1 diabetes. Similarly, Unsworth (14) reported that adaptive bolus calculators leveraging predictive models not only improved glycemic control but also reduced the risk of hypoglycemia in clinical settings. Additionally, meta-analyses by Thomas (15) have highlighted that individualized predictive algorithms can enhance treatment strategies compared to conventional approaches, while multicenter trials, such as that by Beck (16), provide evidence for the clinical efficacy of integrating such models into routine diabetes management. These studies collectively indicate that although predictive models exhibit varying levels of accuracy and clinical impact, they hold significant potential for personalizing diabetes care. Future research should thus aim to refine these algorithms further and rigorously evaluate their integration into everyday clinical workflows, thereby bridging the gap between technological innovation and tangible improvements in patient outcomes.

#### 3.2.9 Limitations

While the bibliometric approach offers a robust quantitative assessment of research trends, several limitations should be acknowledged. First, one key limitation of this study is that the literature search was confined to the Web of Science Core Collection. Although this database is widely recognized for its rigorous indexing criteria and high-quality citation data, relying solely on it may have resulted in the exclusion of relevant studies indexed in other databases, such as Scopus or PubMed, as well as potentially important non-English publications (33, 34). This could limit the comprehensiveness of our analysis and may affect the generalizability of the findings. Future research should consider expanding the data sources to include multiple databases to provide a more exhaustive overview of global research trends in AI-assisted blood glucose management. Second, the exclusion of conference abstracts and other non–peer‐reviewed sources may have led to the omission of some innovative preliminary findings (35). Finally, reliance on citation counts and co–authorship networks does not fully capture the quality or clinical impact of individual studies. Future research could address these limitations by incorporating additional databases and qualitative assessments (35).

#### 3.2.10 Future research directions

The evolving landscape of AI‐assisted blood glucose management suggests several promising avenues for future investigation. Researchers should aim to:

- Validate and refine predictive algorithms: Future studies should leverage larger, multicenter datasets to enhance the generalizability and robustness of AI models (13, 15).
- Integrate novel AI techniques: Exploring advanced methods—such as reinforcement learning (36) and federated learning—could further improve real‐time decision‐making in glycemic control.
- Examine socio–economic and ethical implications: As AI–driven technologies become more prevalent, assessing their impact on healthcare equity and addressing potential disparities is essential (1, 2).
- Foster interdisciplinary collaborations: Bridging clinical expertise and technological innovation remains crucial for translating research findings into practical, patient–centered solutions (37, 38).

## 4 Conclusion

In summary, our bibliometric analysis of AI–assisted blood glucose management provides a detailed mapping of the field’s evolution, highlighting key contributors, research hotspots, and emerging trends (41, 42). The findings demonstrate that AI is not only reshaping diabetes research but is also paving the way for more personalized and effective clinical interventions (9, 13). Continued collaboration and innovation will be essential for overcoming current challenges and harnessing the full potential of AI to improve diabetes care worldwide (39, 40, 43–46).
